# Case Report: ROSAH syndrome presents diagnostic and therapeutic challenges

**DOI:** 10.3389/fopht.2025.1535805

**Published:** 2025-03-25

**Authors:** Jenny Shunyakova, Margaret Reynolds, Amal Taylor, Erin G. Sieck, James T. Walsh, Lynn M. Hassman

**Affiliations:** ^1^ Rocky Mountain Lions Eye Institute, University of Colorado School of Medicine, Aurora, CO, United States; ^2^ John F. Hardesty Department of Ophthalmology and Visual Sciences, Washington University School of Medicine, St. Louis, MO, United States

**Keywords:** ALPK1, ROSAH, optic disc edema, uveitis, retinal degeneration

## Abstract

**Background:**

Retinal dystrophy, optic nerve edema, splenomegaly, anhidrosis, and headache (ROSAH) syndrome is an autosomal dominant disorder caused by a heterozygous missense mutation in alpha kinase 1 (ALPK1). This series reports the presentation and treatment outcomes of three first-degree relatives with ROSAH syndrome.

**Methods:**

Retrospective chart review, whole exome sequencing.

**Results:**

A 16-year-old male presented with bilateral optic disc edema, macular edema, retinal degeneration, and vitreous inflammation. His mother and brother had similar clinical features. Whole exome gene sequencing identified a shared heterozygous mutation in the ALPK1 gene c.710C>T, consistent with ROSAH syndrome. Ophthalmic manifestations in this family included optic nerve edema, macular edema, panuveitis, glaucoma, and widespread retinal cone and rod dysfunction. While the proband’s macular edema improved with intravitreal dexamethasone and systemic tocilizumab, immune suppression did not prevent retinal degeneration.

**Conclusion:**

A diagnosis of ROSAH syndrome, suggested by the concomitant presentation of optic disc edema, uveitis, and retinal degeneration, can be made by targeted genetic sequencing of the ALKP1 gene. While ROSAH-associated ocular inflammation and macular edema may respond to local steroids and immune suppression, retinal degeneration may progress despite these therapies.

## Introduction

Retinal dystrophy, optic nerve edema, splenomegaly, anhidrosis, and headache, known as ROSAH syndrome is a rare, autosomal dominant autoinflammatory disorder, first described in 2012 (1, 2). In this disease, a heterozygous gain-of-function mutation in alpha kinase 1 (ALPK1), a gene that encodes an innate immune receptor for the bacterial sugar, ADP-heptose, results in NFκB–mediated autoinflammation (3). Not surprisingly, patients with this syndrome present with ocular and systemic inflammation, including anterior and posterior uveitis, optic disc and macular edema, recurrent fever, malaise, headaches, arthralgias and abdominal pain, along with cytopenias and elevated inflammatory markers such as C-reactive protein (CRP) and tumor necrosis factor-alpha (TNF-α) (4).

In addition to these inflammatory features, patients with ROSAH syndrome often present with signs of retinal degeneration (5). Optical coherence tomography (OCT) reveals disruption of the retinal pigment epithelial layer and ellipsoid zone (5). Concordantly, full-field electroretinography (ERG) shows reduced scotopic responses, and loss of color vision in the setting of cone or cone-rod dystrophy is also common (5).

ALPK1 is expressed in the optic nerve, retinal pigment epithelium, and the connecting cilium of the photoreceptors, suggesting that the kinase may play a critical role in retinal development or function (6). While the role of ALPK1 in non-immune cell types is poorly understood, it was initially discovered as a key component of apical transport machinery in polarized cells (7). It is not yet known whether the retinal degeneration in ROSAH results from a primary defect in neuronal cells or a consequence of aberrant inflammation.

While the complex pathophysiology of ROSAH is still not fully understood, and the spectrum of clinical manifestations is emerging (4–6), an outstanding question is whether adequately treating ocular inflammation will prevent retinal degeneration. Here we describe the disease course and treatment response of ROSAH due to ALPK1 c.710C>T in one family.

## Case description

### Case 1: Proband

A 16-year-old male was referred to the uveitis clinic for evaluation of vitreous chamber cells and macular edema in the setting of long-standing optic disc edema. He endorsed noticing vision changes from the age of 11 or 12. He was previously diagnosed with idiopathic intracranial hypertension (IIH) and Chiari I malformation at the age of 13, for which he underwent right optic nerve sheath fenestration, ventriculoperitoneal shunt, and was treated with acetazolamide. Despite these interventions, his optic disc edema persisted. The patient also endorsed daily bilateral headaches, arthralgias, lack of sweating, and poor dentition.

**Figure 1:**
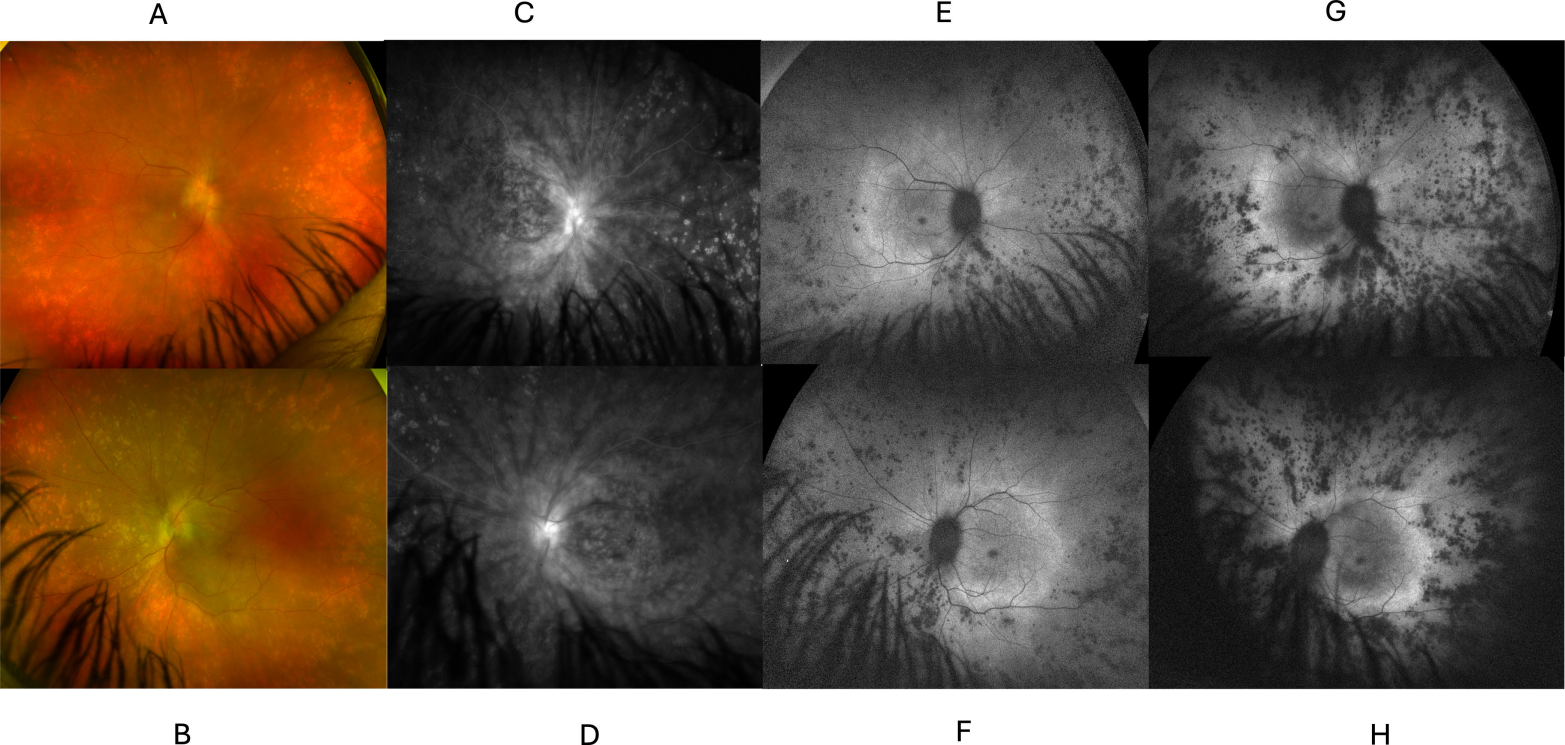
Retinal degeneration concomitant with inflammation of the optic nerve, retinal vessels in the Proband. **(A)** Color fundus photo of the right **(A)** and left **(B)** eyes, demonstrating blurring of optic disc margins, punctate hypopigmented peripheral retinal lesions, and blunted foveal reflex. Fluorescein angiography (FA) of the right **(C)** and left **(D)** eyes demonstrating leakage from the optic disc and proximal vessels, as well as punctate staining peripheral lesions. Fundus autofluorescence (FAF) of the right **(E)** and left **(F)** eyes demonstrating a hyperautofluorescent ring surrounding the macula and diffuse punctate hypoautofluorescent peripheral lesions. **(D)** FAF photos of the right **(G)** and left **(H)** eyes demonstrating progression after 2 years of pigmentary changes and progression on lesions seen in **(E, F)**.

**Figure 2:**
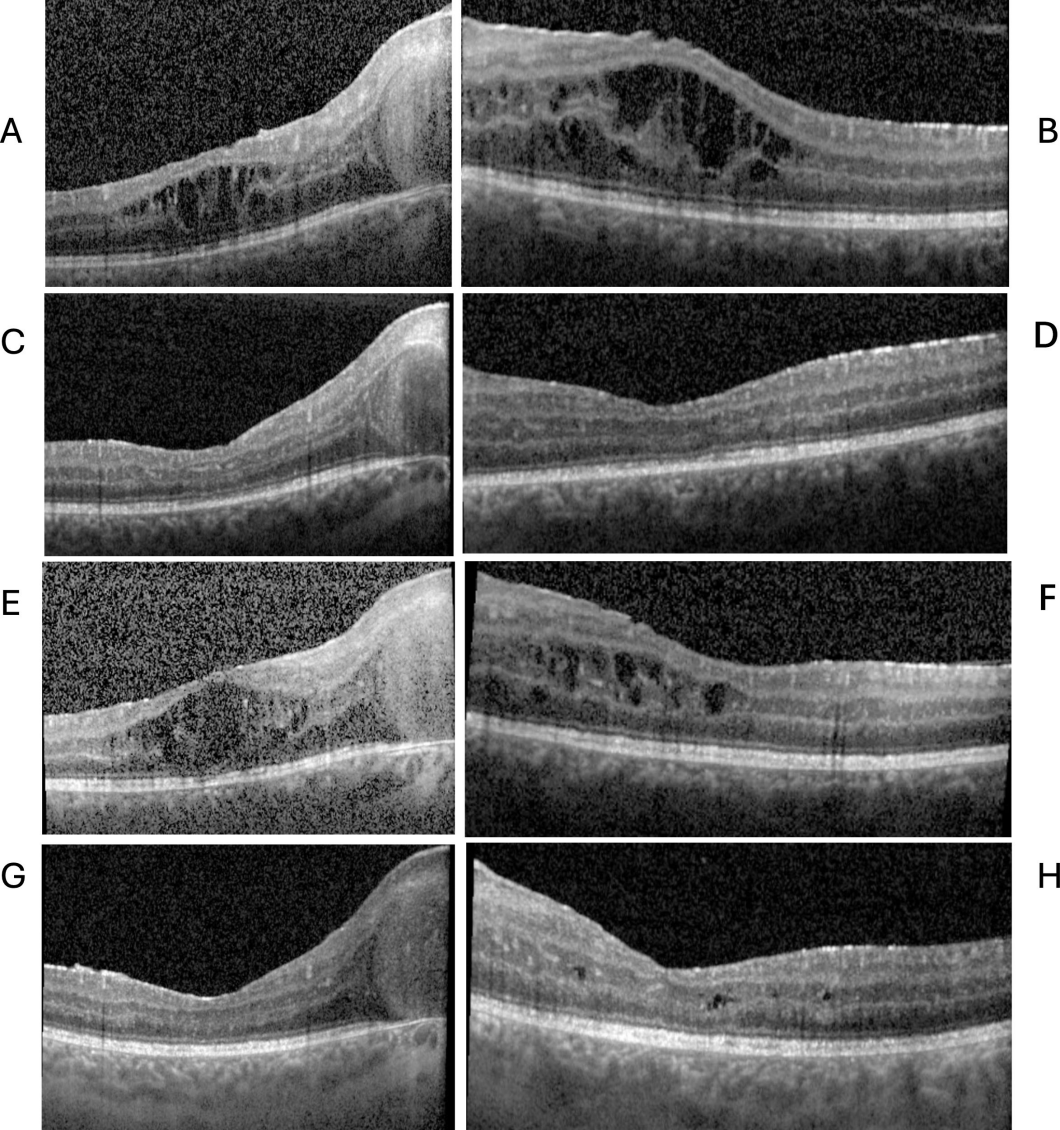
The proband’s macular edema responded to intravitreal dexamethasone and tocilizumab. **(A)** Optical coherence tomography (OCT) of the right **(A)** and left **(B)** eyes demonstrating cystic macular edema on presentation. Improved macular edema of the right **(C)** and left **(D)** eyes after intravitreal dexamethasone injection. Recurrence of macular edema prior to initiation of tocilizumab in the right **(E)** and left **(F)** eyes. Improved macular edema of the right **(G)** and left **(H)** eyes 6 months after initiation of tocilizumab.

At the initial visit, his visual acuity (VA) was 20/90 in both eyes. His intraocular pressure (IOP) was normal. His pupils were normally reactive and there was no afferent pupillary defect (APD). Humphrey visual field showed an enlarged blind spot in both eyes. The right eye had 1+ anterior chamber cells and 2+ vitreous cells with 1+ haze. The left eye had 0.5+ anterior chamber cells and 1+ vitreous cells with 1+ haze. The optic nerves were edematous and gliotic and there was macular edema and hypopigmented spots in the retinal periphery bilaterally. (**Figures 1A, B**) Fluorescein angiography showed significant disc and vascular leakage greatest in the posterior pole, as well as scattered staining spots in the periphery (**Figures 1C, D**). Fundus autofluorescence showed scattered hypoautofluorescent spots peripherally, as well as a hyperautofluorescent ring around the macula (**Figures 1E, F**). Optic coherence tomography (OCT) showed thickened retinal nerve fiber layer and macular edema (**Figures 2A, B**). Electrophysiology revealed widespread cone and rod dysfunction in both eyes (**Supplementary Figure 1**).

The more common infectious and inflammatory causes of uveitis or neuroretinitis were ruled out, as T-Spot, rapid plasma reagin, and treponemal, HIV1/2, myeloperoxidase, proteinase 3, antinuclear, toxoplasma and Bartonella antibodies were all negative, and complete blood count, erythrocyte sedimentation rate and angiotensin-converting enzyme were within normal limits. He was empirically treated with 60 milligrams of daily prednisone, which resulted in minimal improvement in vitritis, disc or macular edema and was tapered over several months. Subsequently, bilateral intravitreal dexamethasone implants resulted in significant improvement in the macular edema (**Figures 2C, D**), but only moderate improvement in vitreous inflammation and minimal change in optic disc edema and vision.

The patient’s mother endorsed a similar clinical history in her teen years, so both the patient and his mother underwent genetic screening with commercially available panel testing for inherited retinal dystrophies. The patient and his mother were both heterozygous for a pathogenic variant in ABCA4 c.6089G>A, p.(Arg2030Gln). Additionally, his mother was heterozygous for 2 potentially pathogenic mutations MYO7A c1895T>G(p.Phe632Cys) and MYO7A c3853 A>C (p.Asn1285His). As these variants did not correlate with the clinical phenotype, the proband, his affected mother and an unaffected grandfather underwent whole exome sequencing, which identified the ALPK1 c.710C>T, p.Thr237Met, mutation shared by both affected patients and not by the unaffected grandfather. The patient was referred to a geneticist for formal diagnosis.

The proband’s macular edema recurred (**Figures 2E, F**) and to spare additional steroids, he was treated with monthly intravenous tocilizumab at a dose of 8 mg/kg, as studies supported it’s efficacy for uveitic macular edema (8), as well as for 2 patients with ROSAH (4). This resulted in significant improvement in the macular edema (**Figures 2G, H**), headaches, and joint pain, however vitreous cell and disc edema persisted. His VA ultimately improved to 20/50 in both eyes. After 5 months of intravenous tocilizumab therapy, the patient missed a dose and was switched to weekly subcutaneous tocilizumab at a dose of 162 mg per week to facilitate better adherence. On this regimen, there was continual improvement in macular edema but the patient’s vitreous inflammation, disc edema, and retinal vascular leakage persisted, and the retinal pigment abnormalities progressed (**Figures 1G, H**). Mycophenolate mofetil 3 g daily and prednisone 10 mg daily were added, however vitreous inflammation and disc edema were persistent and despite 8 months of this combined therapy, he developed anterior chamber inflammation, peripheral anterior synechiae, and elevated IOP of 56 in the left eye necessitating a tube shunt, and the addition of intravitreal dexamethasone and topical difluprednate. Post-operatively his vision was 20/80 in the right eye and 20/200 in the left with a left afferent pupillary defect.

### Case 2: Mother

**Figure 3:**
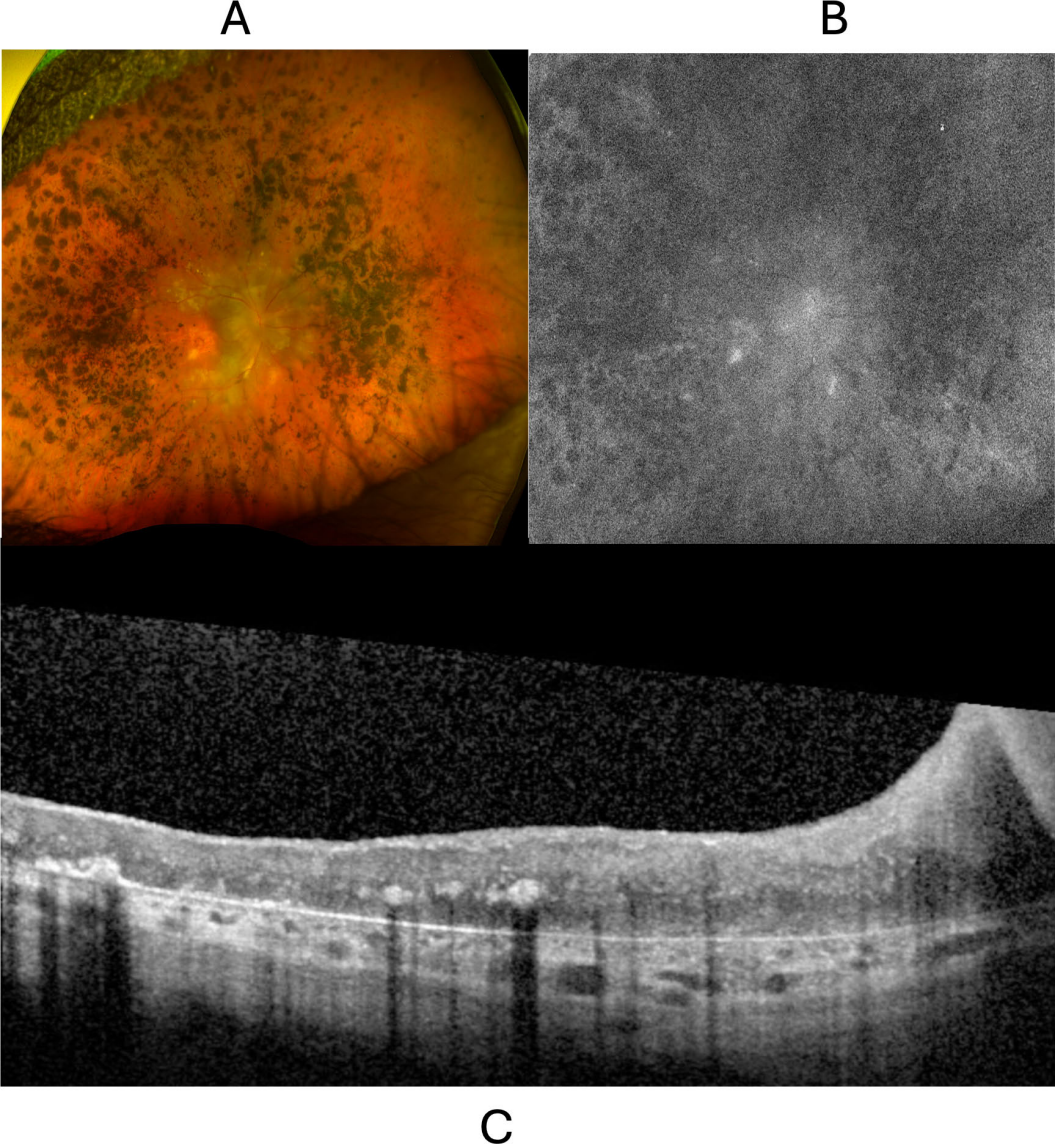
Retinal degeneration in the Proband’s mother. **(A)** Fundus photograph of the right eye showing optic disc gliosis and diffuse punctate hyperpigmented retinal lesions. **(B)** Fundus autofluorescence of the right eye showing hypoautofluorescent punctate retinal lesions. **(C)** OCT of the right eye showing complete outer retinal loss and hyperreflective lesions at the middle retina and retinal pigment epithelial layers, as well as increased hyperreflectivity of Henle fiber layer, consistent with photoreceptor damage (9).

The proband’s mother was a 50-year-old woman with profound vision loss that began when she was 19 and gradually worsened despite multiple therapies and surgeries. She was diagnosed with IIH at age nine. No medical records were available, however she described a similar presentation. She was diagnosed with polyarthritis, and reported complete anhidrosis. Like her son, she underwent optic nerve fenestration with no alleviation of optic nerve edema. She was subsequently treated with high-dose prednisone, azathioprine and cyclophosphamide for presumed uveitis, also with no alleviation of her ocular inflammation. Despite these therapies, her visual acuity declined to light perception during her twenties, and she stopped seeing ophthalmology. On exam at our clinic, her VA was bare light perception in both eyes. Slit lamp exam showed bilateral iridodonesis and aphakia, but no anterior chamber cells. There was 1+ vitreous cells without haze in both eyes. Fundus examination showed hyperpigmented lesions in the peripheral retina, retinal vascular attenuation, and gliosis and pallor of the optic discs (**Figures 3A, B**). OCT revealed loss of the outer nuclear and outer plexiform layers along with Henle fiber layer reflectivity indicative of photoreceptor damage (9) (**Figure 3C**). ERG of the mother was not performed. Due to previous inefficacy of therapy, the patient was not interested in treatment.

### Case 3: Younger brother

**Figure 4:**
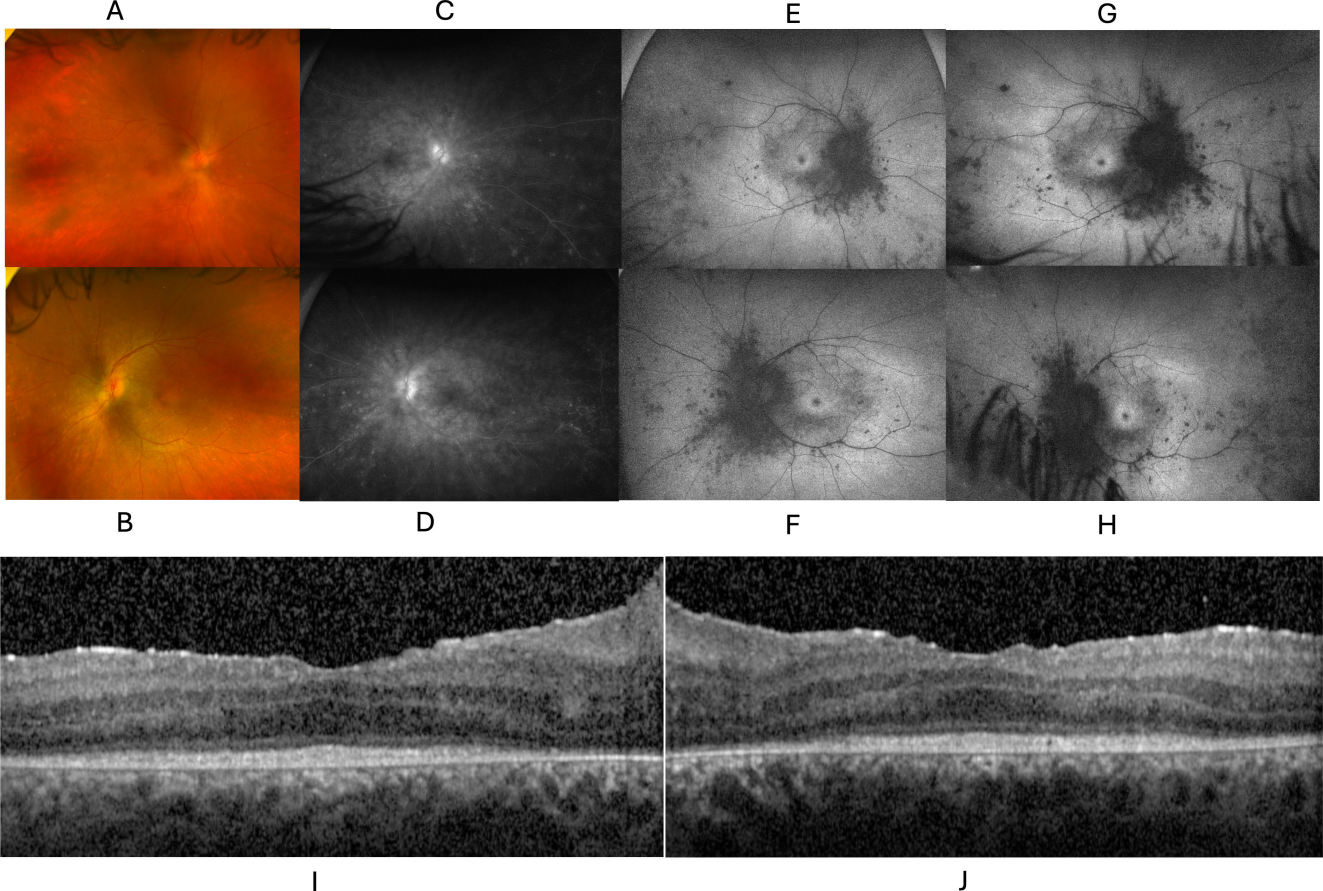
Retinal degeneration concomitant with inflammation of the optic nerve, retinal vessels in the Proband’s brother. Color fundus photo of the right **(A)** and left **(B)** eyes, demonstrating blurring of optic disc margins, punctate hypopigmented peripheral retinal lesions, and blunted foveal reflex. Fluorescein angiography (FA) of the right **(C)** and left **(D)** eyes demonstrating leakage from the optic disc and proximal vessels, as well as punctate staining peripheral lesions. Fundus autofluorescence (FAF) of the right **(E)** and left **(F)** eyes demonstrating a hyperautofluorescent ring surrounding the macula and diffuse punctate hypoautofluorescent peripheral lesions. **(D)** FAF photos of the right **(G)** and left **(H)** eyes demonstrating progression after 2 years of pigmentary changes and progression on lesions seen in **(E, F)** OCT of the right **(I)** and left **(J)** eyes showing outer retinal loss without macular edema.

The proband’s younger brother later presented to the uveitis clinic at age 16 with blurry vision. He noted progressive blurring of vision, trouble with night vision, trouble adjusting from bright lights to dim lights, and intermittent episodes of pressure sensation accompanied by darkening of his peripheral vision since the age of 15. He denied any migraine headaches, joint pains, or anhidrosis. His visual acuity was 20/40 in both eyes. There was no APD, and his confrontation visual fields were full bilaterally. Both eyes had bilateral trace anterior chamber cells, 0.5+ cells and 1+ haze in the vitreous, edematous optic discs, scattered hypopigmented spots in the posterior poles and epiretinal membranes and outer retinal disruption (**Figure 4**). ERG showed severe widespread cone and rod dysfunction in both eyes (**Supplementary Figure 2**).

He was initially unwilling to receive medical therapy until he presented with acute eye pain and loss of vision. The left eye had developed 0.5+ anterior chamber cell, with corneal edema and elevated intraocular pressure of 56 with open iridocorneal angles despite scattered peripheral anterior synechiae. He was treated with oral prednisone and topical prednisolone and underwent Ahmed valve placement.

The patient was unwilling to take injected therapies, so he started on oral tofacitinib at a dose of 11 mg daily, with the rationale that broad inhibition of cytokine signaling, including IL-6 might be beneficial. Despite continuing this therapy for two months, the right anterior chamber inflammation and disc and vascular leakage persisted, thus he started topical difluprednate in addition to tofacitinib. Two months later, the patient had stopped his medications, however, visual acuity was stable at 20/60, and IOP was normal in both eyes.

This patient did not undergo genetic testing due to financial constraints, but the similarity of his presentation to his mother and brother suggested a common etiology. **Supplementary Figure 3** demonstrates a pedigree of affected individuals in the family.

## Discussion

Here we describe a family with a shared set of clinical features and an ALPK1 c.710C>T mutation, consistent with ROSAH syndrome. All three of our patients experienced loss of vision in the second decade of life associated with optic disc edema. Additionally, the two patients who underwent ERG testing had widespread cone and rod dysfunction. Consistent with the family in this series, the most common ophthalmic feature of ROSAH is decreased visual acuity associated with optic nerve edema. This feature has been noted in patients as early as 4 years old and was present by age 12 in all cases in the first reported series (6). Disc edema was such a prominent feature in our cases that both the proband and his mother were initially misdiagnosed with idiopathic intracranial hypertension and underwent optic nerve sheath fenestration.

Other frequently observed features include inflammation of the anterior and vitreous chambers, retinal vasculitis, retinal degeneration, macular edema, and glaucoma (5). Two of our three patients developed glaucoma associated with active anterior segment inflammation and peripheral anterior synechiae.

Commonly reported extra-ophthalmic manifestations include elevated CRP, pancytopenia, anhidrosis, idiopathic massive splenomegaly, migraine headaches, arthralgias, and joint deformities. Patients may have premature mineralization and calcification of the basal ganglia (4), and meningeal enhancement suggesting that central nervous system inflammation is also a feature of this disease (4). Dental abnormalities are also common and involve both the root and enamel (4).

The most commonly described ALPK1 variant in patients with ROSAH syndrome is c.710C>T, p.Thr237Met, although c.761A>G, p.Tyr254Cys (Y254C) (4), and c.830 C>T, p.Ser277Phe (10) have also been described. ALPK1 is a pattern recognition receptor, serving as a cytosolic sensor for bacteria-derived metabolites, including ADP-heptose, an intermediate carbohydrate metabolite of the lipopolysaccharide (LPS) biosynthesis pathway, which triggers the activation of the NF-kB pathway and secretion of inflammatory cytokines by immune cells (3, 7). In support of the role of ALPK1 in activating inflammation in ROSAH, Kozycki et al. found that 7/10 patients treated with anti-cytokine therapy experienced some improvement in their symptoms (4).

IL-6 is a pivotal cytokine in retinal inflammation, influencing downstream pathways that impact immunologic tolerance and inflammation (11). Notably, the IL-6 inhibitor, tocilizumab, is effective at reducing macular edema associated with uveitis (12) and may also be beneficial in treating macular edema in the setting of retinal degeneration (8). Consistent with this, in a series of ROSAH patients from multiple countries, tocilizumab improved macular edema and/or retinal vascular leakage in 2 cases, whereas inhibitors of TNF-α and IL-1β were generally less effective at treating ocular manifestations (4). Likewise, our proband’s macular edema resolved after both intravitreal dexamethasone and systemic tocilizumab, although oral prednisone was ineffective. In both the proband and his brother, uncontrolled uveitis led to glaucoma. These cases demonstrate the potential for vision-threatening sequelae in ROSAH if ocular inflammation is not adequately controlled.

It is also noteworthy that anti-inflammatory therapy did not prevent progressive pigmentary retinopathy in our proband or severe loss of vision in his mother. While earlier and/or more aggressive therapy may have abrogated retinal degeneration in our cases, the etiology of retinal degeneration in ROSAH may be independent of inflammation. Retinal-specific dysfunction in ALPK1 was suggested by Williams et al (6). Consistent with the notion of a role for ALPK1 outside of inflammation, several other clinical manifestations of ROSAH, including dental hypoplasia and anhidrosis are also not typically attributed to inflammation (3).

ALPK1 is broadly expressed by multiple tissues and is located within cells at the centrosome, a microtubule-organizing organelle critical for mediating cell division, polarity, and migration. ALPK1 plays a role in apical membrane transport by phosphorylating myosin (7), and cancer-associated mutations drive cell migration, invasion, and proliferation- all independent of inflammation (13). Within the retina, ALPK1 is localized to the basal body centrioles of the photoreceptors, which are modified cilium, and also in the retinal pigment epithelium (RPE) (7). Phototransduction requires a tremendous flux of intracellular materials, and dysfunctional cilia and ciliary-centrosomal abnormalities, as well as defective RPE polarity, can lead to retinal degeneration associated with systemic ciliopathies, such as Bardet-Biedl syndrome (14). The subcellular localization of ALPK1 suggests that retinal degeneration in ROSAH may also represent a form of ciliopathy. Likewise, similarities between the dental anomalies associated with ROSAH and with primary ciliopathies (15) suggest a role for dysfunctional ciliary ALPK1 in the dental epithelium and mesenchyme.

Our proband and his mother also expressed heterozygous c.6089G>A variant in ABCA4. Similarly, compound heterozygous ABCA4 variants (c.2366G>C; (p.Gly863Ala) and c.5882G>A; (p.Gly1961Glu)) were reported in one patient with ROSAH with an ophthalmic phenotype similar to our patients (16). More than 800 variants in ABCA4 have been associated with a spectrum of retinal disease, in which cumulative loss of function is hypothesized to be associated with more severe retinopathy (17). It is therefore possible that the co-expression of disease-associated ABCA4 variants may compound the retinal dysfunction seen in ROSAH. Additionally, two potentially pathogenic variants in MYO7A were detected in only the mother. While she did not have hearing loss associated with mutant MYO7A in Usher syndrome (18), given the critical role of MYO7A in the retina (19) these variants could have contributed to her retinal degeneration.

In conclusion, ROSAH syndrome is an underrecognized clinical entity. When faced with coincident uveitis and retinal degeneration, clinicians should consider obtaining a family pedigree and performing targeted sequencing to detect mutations in ALPK1. While some features of the disease likely result from aberrant inflammation triggered by mutant ALPK1, other features including retinal degeneration are poorly understood. Clinical features such as macular edema and joint pain may respond favorably to anti-inflammatory therapies like IL-6 inhibitor, tocilizumab, however, long-term follow-up is necessary to determine whether these therapies will ameliorate retinal degeneration and other disease manifestations.

Further research is needed to understand how mutations in ALPK1 lead to the disease manifestations so that highly effective treatments can be developed.

## Data Availability

The original contributions presented in the study are included in the article/**Supplementary Material**, further inquiries can be directed to the corresponding author/s.
