# Supplementary material for: Case Report: ROSAH syndrome presents diagnostic and therapeutic challenges
Source: Front Ophthalmol (Lausanne). 2025 Mar 25;5:1535805. doi: 10.3389/fopht.2025.1535805 (PMC11975653; doi:10.3389/fopht.2025.1535805)
Supplement: Supplementary file 2 [file DataSheet2.pdf]

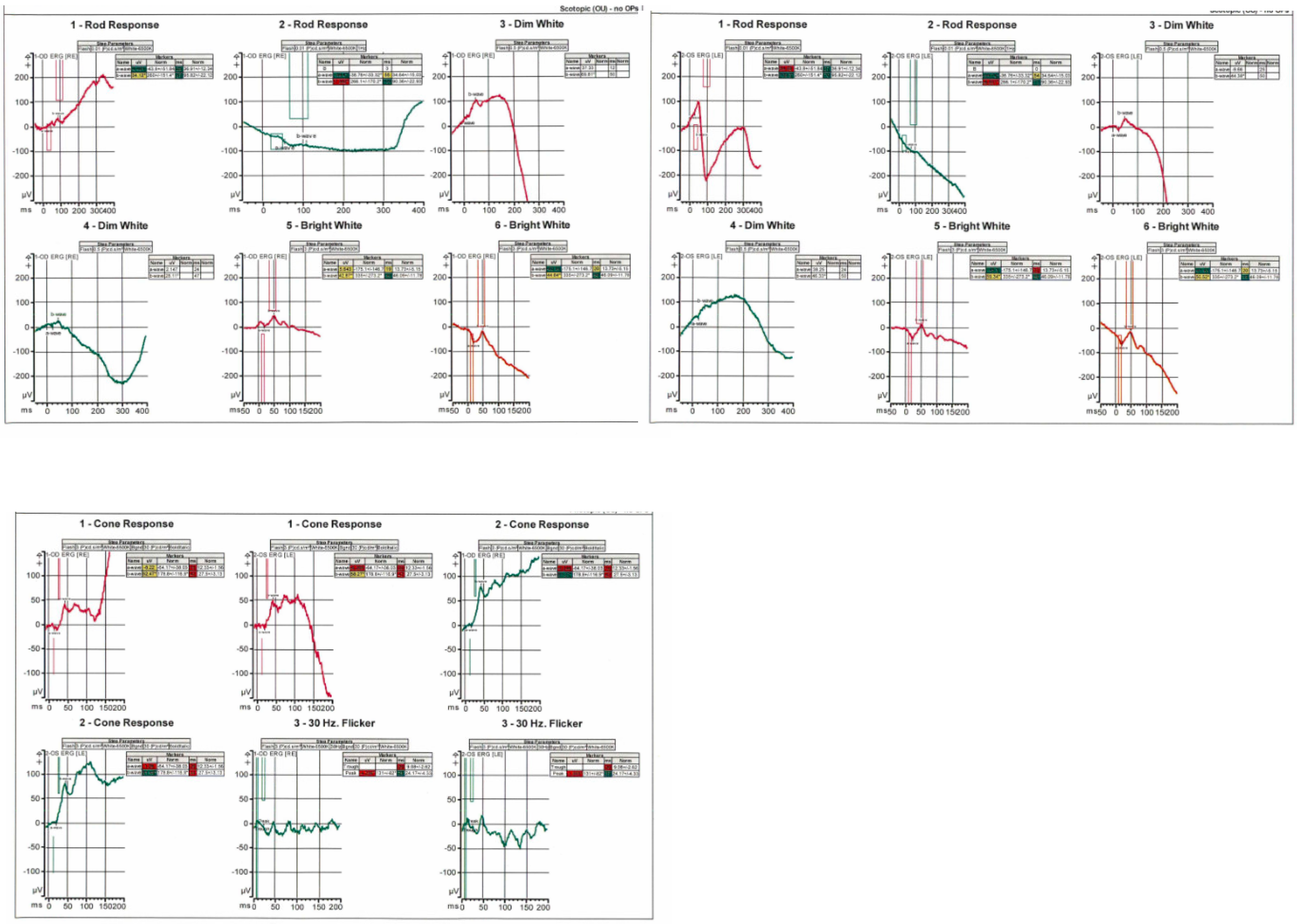

Supplemental Figure 2: ERG demonstrates widespread bilateral cone and rod dysfunction in the proband's brother.
