# Supplementary material for: Case Report: ROSAH syndrome presents diagnostic and therapeutic challenges
Source: Front Ophthalmol (Lausanne). 2025 Mar 25;5:1535805. doi: 10.3389/fopht.2025.1535805 (PMC11975653; doi:10.3389/fopht.2025.1535805)
Supplement: Supplementary file 3 [file DataSheet3.pdf]

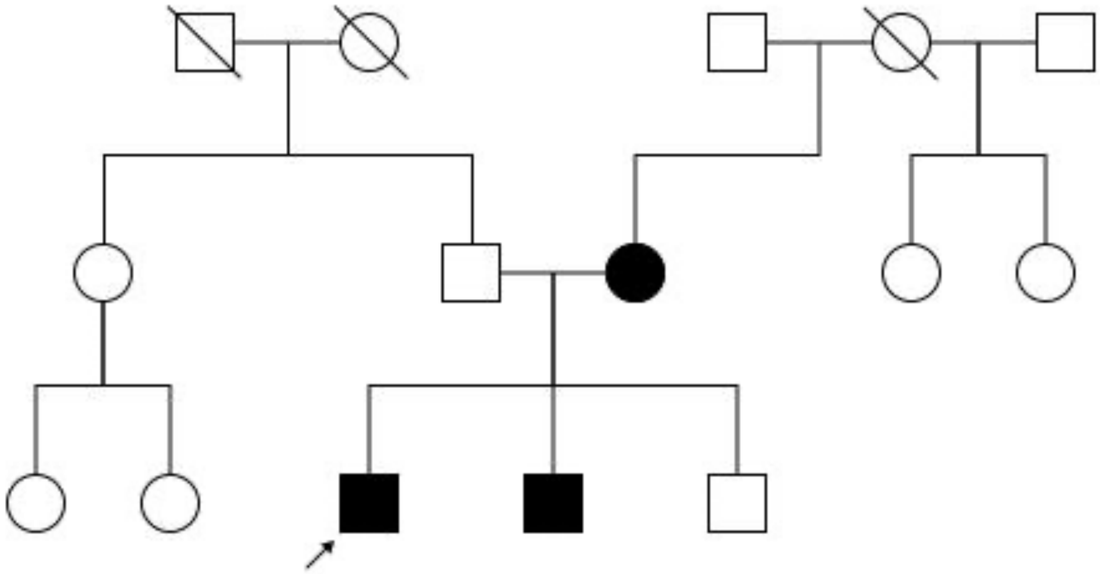

**Supplemental Figure 3: Family Pedigree demonstrating affected proband (arrow), his brother and mother.**
